# Is It the pH That Matters? Challenging the Pathophysiology of Acidemia in a Case of Severe Hypercapnia Secondary to Intraoperative CO_2_ Insufflation

**DOI:** 10.1155/2020/1898759

**Published:** 2020-09-27

**Authors:** Evan Merle, Saad Zaatari, Rory Spiegel

**Affiliations:** ^1^Department of Surgery, MedStar Washington Hospital Center, Georgetown University School of Medicine, Washington, DC, USA; ^2^Department of Emergency Medicine, MedStar Washington Hospital Center, Georgetown University School of Medicine, Washington, DC, USA; ^3^Department of Emergency Medicine and Department of Critical Care Medicine, Medstar Washington Hospital Center, Georgetown University School of Medicine, Washington, DC, USA

## Abstract

**Background:**

Acidemia has been long thought to lead to hemodynamic compromise. While some literature to date challenges this idea, there is no consensus on this topic.

**Case Summary:**

To our knowledge, this is the most severe case of hypercapnia and acidosis due to carbon dioxide (CO_2_) insufflation during laparoscopy reported in the literature. Remarkably, this patient remained hemodynamically normal despite having a blood pH below 6.81. This prompts a wider discussion about the effects of blood pH on human physiology. Most patients who present acidotic are critically ill and have confounding underlying metabolic or respiratory pathophysiology driving their illness. In this case, the patient experienced no respiratory insult leading to an increase in blood CO_2_ but rather had CO_2_ iatrogenically introduced into the circulatory system, effectively detaching the deleterious effects of CO_2_ from the respiratory pathologies that so often cause its accumulation.

**Conclusion:**

This raises the question, in patients with severe acidosis and hemodynamic compromise, is acidosis a symptom of the underlying process, or is the acidosis itself causing harm?

## 1. Background

Carbon dioxide (CO_2_) is the preferred gas for laparoscopic procedures for a number of reasons. This gas has a superior safety profile because it does not support combustion and has a lower risk of gas embolism [1]. It also has a high diffusion coefficient and high solubility in blood and is a normal metabolic end product: properties that lend to it being rapidly cleared from the body [1]. Nevertheless, hypercapnia, or a rise in blood CO_2_, is a known association with the use of carbon dioxide insufflation during laparoscopic surgery. To date, all case reports describing this phenomenon have reported modest increases in partial pressures of carbon dioxide (PaCO_2_). We present a case of severe hypercapnia and acidemia secondary to carbon dioxide insufflation during a laparoscopic paraesophageal hernia repair. This case prompts a wider discussion about the implications of increased PaCO_2_ and acidemia on human physiology and hemodynamics. In particular, it questions the commonly held belief that severe acidemia leads to hemodynamic collapse.

## 2. Case Report

We present the case of an 83-year-old female with a past medical history of hypertension, hyperlipidemia, and hypothyroidism who presented to the emergency department with back pain, nausea, and vomiting. A large retro cardiac paraesophageal hernia (type III) was diagnosed on computed tomography. She was scheduled for an elective laparoscopic paraesophageal hernia repair due to persistent severe symptoms, weight loss, and the risk for volvulus. Intraoperatively, no major difficulties or complications were noted by the surgical team. Towards the end of the case, the anesthesia team observed an unexpected increase in end-tidal CO_2_ levels. An arterial blood gas (ABG) indicated a pH of <6.81 with a PaCO_2_ that was incalculably high (max measurable value is 115 mmHg). The partial pressure of oxygen (PaO_2_) was 118 mmHg on a fraction of inspired oxygen (FiO_2_) of 40% and positive end-expiratory pressure (PEEP) of 5 cm H_2_O. The bicarbonate (HCO_3_) could not be calculated due to severe acidosis. The ionogram was normal in this sample. Peak and plateau pressures were 28 cm H_2_O and 19 cm H_2_O, respectively. Interestingly, the patient remained hemodynamically normal despite this profound acidemia and did not require any vasopressors or intravenous fluid boluses.

**Figure 1:**
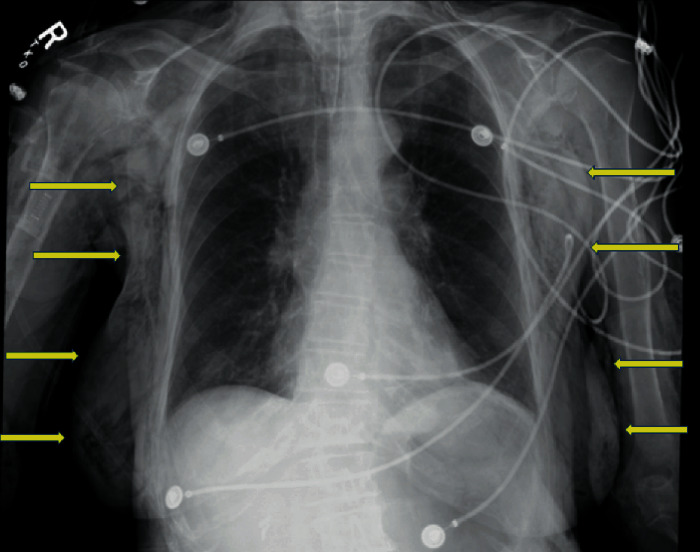
CXR with arrows demonstrating severe subcutaneous emphysema.

The patient remained intubated, deeply sedated, and was taken to the intensive care unit for the management of severe respiratory acidosis. The ventilatory changes included increasing the respiratory rate from 12 to 20 breaths per minute and the tidal volume from 400 mL to 450 mL. Repeat ABG after one hour showed a pH 7.22, PaCO_2_ 58 mmHg, PaO_2_ 166 mmHg, and HCO_3_ 23.7 mEq/L. A significant degree of crepitus was noted tracking along the chest, neck, arms, and hands (Figure 1). This prompted suspicion for diffuse subcutaneous gas due to CO_2_ insufflation and passive absorption of CO_2_ causing acidosis. A third ABG, two hours after the first, showed an overcorrection of the hypercarbia with pH 7.48, PaCO_2_ 28 mmHg, PaO_2_ 171 mmHg, and HCO_3_ 20.9 mEq/L. At this point, the respiratory rate was decreased to 12 breaths per minute with no other changes on the ventilator, and an ABG two hours later revealed pH 7.42, PaCO_2_ 34 mmHg, PaO_2_ 174 mmHg, and HCO_3_ 22.1 mEq/L. The patient was extubated the following morning requiring only 2 L/min via nasal prongs. The patient's hospital course was complicated by a contained esophageal leak, and after successful treatment, she was eventually discharged to subacute rehabilitation.

## 3. Discussion

Hypercapnia and acidosis are well-known complications of laparoscopic surgery related to abdominal CO_2_ insufflation. Typically, hypercapnia occurs via two discrete mechanisms: the gas is either directly absorbed into the circulation, or it develops secondarily to decreased cardiopulmonary ventilation and decreased venous return from the elevated intra-abdominal pressure from insufflation. When hypercapnia is severe, it is frequently associated with extensive subcutaneous emphysema, as was seen in this case, which allows for a greater overall tissue surface area of absorption, and thus a more severe and prolonged acidosis [2].

Overall, subcutaneous emphysema is a relatively common finding after laparoscopic procedures, with prior reviews reporting incidence as high as 3%. Causes can vary; however, it is associated with prolonged operative times, repeated attempts at obtaining insufflation, increased gas volume or flow rate, excessive torque on trocar sites, and the number of trocar sites used (risk increases with additional sites). Thus, the primary mechanism is gas dissecting through tissue planes via defects in the fascia and peritoneum created by trocar entry [2]. This in addition to the risk of air escaping the peritoneal cavity during upper abdominal surgery, which at times may allow for a direct extension of gas to the mediastinum and pleural spaces through the diaphragmatic hiatus. Both mechanisms ultimately allow for a greater surface area of gas absorption and additional risk of subcutaneous gas expansion through tissue planes.

An early case report published by Kent in 1991 documented a pH of 7.06 and PaCO_2_ of 104 mmHg after an elective laparoscopic cholecystectomy and appears to be one of the most severe elevations reported to date [3]. Additional case studies documenting subcutaneous emphysema in cases ranging from laparoscopic cholecystectomies to laparoscopic gynecologic procedures have demonstrated similar findings of increased EtCO_2_ and supposed hypercapnia [4, 5]. In a case report by Coronil et al. presenting a case of a laparoscopic hemicolectomy, an ABG study sent following marked subcutaneous emphysema and increased ETCO_2_ showed a pH of 7.16 and PaCO_2_ of 76.7 mmHg, again less pronounced findings than the case we present here. Additionally, the hemodynamic change they encountered was hypertension requiring medical therapy as opposed to any evidence of distributive shock or vasodilatory reaction to acidosis [6].

The patient described in this report did not have more serious sequelae sometimes seen with subcutaneous emphysema after laparoscopy, such as CO_2_ air embolism or clinically significant pneumothorax. Chest X-rays may miss anterior pneumothoraces, and our patient did not have a repeat chest CT immediately postoperatively; however, plateau pressures remained normal at approximately 20 cm H_2_O while the patient was intubated, and daily chest X-rays for 3 days after her surgery did not show any evidence of a pneumothorax. From a physiologic and metabolic perspective, her primary issue was a severe acidosis, which overall did not appear to impact her hemodynamic status.

This case report brings up the obvious question, does the degree of acidosis, the quantity of hydrogen ions in the serum, and the pH matter? Or is it the underlying etiology which leads to the hemodynamic perturbations so often associated with severe acidemia? The idea that severe acidosis, within itself, has deleterious effects on hemodynamics and needs to be fixed is a common belief among critical care physicians [7]. A major prevailing theory is acidosis impairs myocardial function leading to refractory hypotension, decreased responsiveness to pressors, and ultimately multiorgan failure [8]. While this seems physiologically plausible, there is a paucity of data that identifies the acidosis itself to be the problem.

Studies examining this question are often confounded by the fact that it is almost impossible to separate the effects of the acidemia from the effects of the underlying disease state. In the case of acidemia due to hypercapnia, it almost always occurs under clinical circumstances in which the patient is experiencing some form of respiratory compromise. Given this, it is often difficult to determine what proportion of the stress experienced by the patient is due to the acidosis versus the underlying respiratory pathology. Our case offers a glimpse into the true effects of hypercapnia in isolation. In this case, the patient experienced no respiratory insult leading to an increase in pCO_2_ but rather had CO_2_ iatrogenically introduced into the circulatory system, effectively detaching the deleterious effects of CO_2_ from the respiratory pathologies that so often cause its accumulation.

This is not the first report to document the lack of hemodynamic decompensation observed in cases of severe acidemia due to hypercapnia. Frumin et al. demonstrated patients tolerated acidosis from hypercapnia to extremely low levels (pH as low as 6.8) without any signs of physiological distress or hemodynamic compromise [9]. In a randomized crossover study, Mas et al. investigated the effects of moderate hypercapnia on the hemodynamic parameters of 10 mechanically ventilated patients by varying instrumental dead space. Acute moderate hypercapnia, defined as an average increase in PaCO_2_ from 40 to 52, was associated with mild increases in heart rate and cardiac index, while systemic vascular resistance decreased [10]. Assuming a similar trend with increasing hypercapnia, these hemodynamic changes would be expected to be associated with increased tissue perfusion rather than hemodynamic collapse.

This phenomenon is not isolated to hypercapnia. Olympic rowers, shortly after a race, have demonstrated elevations in serum lactate values as high as 24 mmol/L, leading to pHs as low as 6.8 without any ill effects [11]. A recent article in Critical Care Medicine by Masevicius et al. supports these experiences. In a prospective observational cohort, the authors enrolled 4901 ICU patients, 1609 meeting the criteria for metabolic acidosis. The authors reported that for any given pH, acidosis due to lactate or unmeasured anions had a much higher mortality than those due to hyperchloremia, which demonstrated mortality rates similar to patients without acidosis [12]. Conversely, several studies examining therapies aimed at correcting the serum pH in patients presenting with severe acidosis have consistently failed to demonstrate improvements in patient-centered outcomes [13–15].

Beyond hemodynamics, acidosis and hypercapnia have been shown to affect other aspects of human physiology. For example, hypercapnic respiratory acidosis has been postulated to cause cerebral vasodilation via perivascular extracellular pH changes; however, this usually only leads to cerebral edema in the presence of an acute brain injury [16]. Hypercapnic acidosis has also been thought to increase pulmonary vascular resistance which can be particularly detrimental in patients with preexisting pulmonary hypertension and infants [17]. In the above described clinical scenario, we did not specifically look at markers of cerebral edema or increased pulmonary vascular resistance; however, we argue if present, they were clinically inconsequential as it did not seem to affect her clinical course. These potential effects may have been more profound in a different clinical scenario where insults to the central nervous or pulmonary systems were present. Furthermore, this case describes a brief episode of severe acidosis without any significantly associated sequelae and does not speak to the potential effects of prolonged acidosis.

## 4. Conclusions

The case report we have presented above described a severe acidemia below <6.81, a level at which traditionally has been thought to not be compatible with life, in a hemodynamically stable patient. In this case, the underlying cause was benign. This case offers another piece of evidence that the serum pH may be just a number, not an independent marker or predictor of outcomes.
